# Implementation of Telemedicine for Patients With Dementia and Their Caregivers: Scoping Review

**DOI:** 10.2196/65667

**Published:** 2025-05-05

**Authors:** Mengfei Ye, Zheng Liu, Weigen Xie, Mengna Shou, Shengpang Wang, Xuebing Lin, Yan Xu, Miner Yao, Jialu Chen, Yunli Shou, Jingzhu Wu, Lili Guan

**Affiliations:** 1 Department of Psychiatry, Shaoxing Seventh People's Hospital, Affiliated Mental Health Center of Shaoxing University Shaoxing China; 2 Department of Pharmacology, School of Medicine, Shaoxing University Shaoxing China; 3 Department of Women's Health, Shaoxing Maternity and Child Health Care Hospital Shaoxing China

**Keywords:** telemedicine, dementia, mobile phone, implementation, telehealth, scoping review, family caregiver, systematic search, mental health, mental illness, mental disorder

## Abstract

**Background:**

As dementia advances, symptoms and associated concerns lead to significant distress for both the patients and their caregivers. Telemedicine has the capacity to alleviate care-related issues for patients with dementia and their family caregivers.

**Objective:**

This study aims to synthesize the implementation strategies for providing telemedicine to assist patients with dementia and their caregivers in home and community settings and to examine its effectiveness and implementation barriers.

**Methods:**

In accordance with the PRISMA-ScR (Preferred Reporting Items for Systematic Reviews and Meta-Analyses extension for Scoping Reviews) guidelines, a systematic search was conducted across 6 databases, including MEDLINE, Embase, PsycINFO, CINAHL, Web of Science, and ProQuest. The eligibility criteria for peer-reviewed English publications from January 2000 up to January 2025 encompassed research studies related to telemedicine services for individuals diagnosed with dementia and caregivers.

**Results:**

This study included 54 articles, comprising 14,446 participants from 10 countries. In total, 4 major themes emerged from the articles: the design of telemedicine services, effectiveness of telemedicine, external environmental factors, and barriers in telemedicine implementation. Cognitive training was addressed in 28 studies. Within the domain of work and leisure, 24 solutions were identified. Most reviewed studies indicated favorable experiences with telemedicine services and highlighted perceived personal and social benefits among patients with dementia, as well as identified barriers to accessing and using such services.

**Conclusions:**

Future studies should encompass the enhancement of digital accessibility for individuals with restricted resources and limited technological proficiency, the use of randomized controlled trial methodologies to ascertain the comparative efficacy of various service delivery modes, and the augmentation of sample diversity.

## Introduction

Dementia is a complex neurodegenerative condition characterized by progressive deterioration and a diverse array of subtypes and clinical manifestations. According to a recent report, there will be an estimated 152.8 million cases of dementia worldwide by the year 2050, posing a significant burden on health systems that are already facing challenges in meeting the needs of the elderly population [1]. As dementia progresses, the care needs become more complex. In order to effectively fulfill their responsibilities, caregivers require support to mitigate heightened levels of stress, anxiety, burnout, and depression that often result from the demands of caregiving [2].

In light of the COVID-19 pandemic, nations proactively implemented preventive strategies, including the prohibition of large gatherings, the suspension of entry for travelers from specific regions, and the limitation of face-to-face medical services [3]. The pandemic had a serious impact on older individuals with dementia and their families [4]. A study conducted from March to December 2020 revealed a 25.7% increase in the mortality rate among individuals living with dementia compared to the corresponding period in the previous year, representing nearly twice the rate observed in the elderly population without dementia [5]. However, historical evidence suggests that the occurrence of new pandemics or other disasters is inevitable [6].

Health care interventions for patients with dementia are increasingly incorporating technology to address the escalating costs of health and social care, as technology-based interventions require fewer staff and can reach a larger number of individuals at a comparable cost. The acceleration of this transition can be attributed to the impact of the COVID-19 pandemic, prompting numerous services to shift to a digital format [7]. Telemedicine is defined as the use of internet-connected devices such as computers, tablets, or smartphones to facilitate communication between health care providers and patients [8]. This form of health care delivery can be classified as either synchronous, involving real-time interactions, or asynchronous, involving delayed communication through various mediums such as data, images, messages, or prerecorded videos [9]. Numerous services were already accessible via Telemedicine for purposes such as clinical consultation, diagnosis, evaluation, and psychotherapy [10]. Telemedicine has the potential to aid health care providers in efficiently diagnosing individuals with dementia and their caregivers, especially those living in rural areas [11]. However, the field of telemedicine research faces various challenges, such as people with dementia struggling to focus and communicate with health care providers digitally.

We described and synthesized the implementation strategies used for the provision of Telemedicine to assist patients with dementia and caregivers in home and community settings. The secondary aim was to identify the effectiveness and barriers to the implementation of telemedicine.

## Methods

This scoping review was carried out in accordance with the PRISMA-ScR (Preferred Reporting Items for Systematic Reviews and Meta-Analyses extension for Scoping Reviews) guidelines [12].

### Selection Criteria

We conducted a comprehensive search of articles from January 2000 to January 2025 across 6 databases, including MEDLINE, Embase, PsycINFO, CINAHL, Web of Science, and ProQuest. The literature search was limited to studies published from 2000 onward, as telemedicine for patients with dementia gained significant attention with technological advancements and increasing recognition of its role in health care access [13]. Before 2000, research in this area was still emerging, and telemedicine interventions were less commonly studied or implemented. This time frame ensures the inclusion of the most relevant and impactful studies in this evolving field. Preprint servers were excluded from the search strategy due to their absence of peer review and quality control mechanisms. The search strategy was formulated across 3 core domains, namely, dementia, caregivers, and telemedicine (Tables S1 and S2 in the Multimedia Appendix). We conducted an initial limited search on PubMed to identify pertinent keywords and MeSH (Medical Subject Headings) terms within the 3 domains. Subsequently, we used various combinations of these terms to search across multiple databases. To conduct a thorough search, we examined the reference lists of all articles that were included in the study to uncover additional relevant studies.

### Eligibility Criteria

We exclusively reviewed English publications in peer-reviewed journals without restricting the types of publications, thereby encompassing original research articles, reports, and reviews for screening. The studies included in this analysis must pertain to telemedicine services for dementia or cognitive impairment, as well as family caregivers who assist individuals with dementia or cognitive impairment, or both populations. The participants were not limited to individuals with dementia or caregivers; perspectives were also sought from telemedicine service providers and other pertinent stakeholders.

### Selection of Studies

Two authors (MY and FH) independently evaluated all articles that emerged from the search strategy. Initially, the titles and abstracts were reviewed to assess relevance. For entries deemed potentially relevant, the full texts were thoroughly examined by both reviewers to confirm their pertinence to the research question. Any discrepancies in article selection were resolved through consensus meetings between the 2 reviewers.

All relevant articles were thoroughly reviewed and coded to identify recurring patterns or concepts. These codes were grouped into categories based on similarities. Through several rounds of discussion and refinement, the authors identified 4 major themes: design of telemedicine services, effectiveness of telemedicine, external environmental factors, and barriers in telemedicine implementation. The final themes were reached through consensus among the authors to ensure accuracy and consistency.

### Data Extraction and Data Analysis

Two authors (MY and XL) independently gathered data from the articles that were included. In accordance with the CONSORT-EHEALTH (Consolidated Standards of Reporting Trials of Electronic and Mobile Health Applications and Online Telehealth) guidelines [14], the data extracted from the selected articles encompassed various categories, including article attributes (such as authorship, publication date, and country of origin), patients with dementia (including diagnosis and age), caregiver demographics (including age, gender, and educational background), study parameters (such as theoretical underpinnings, research methodology, objectives, participant selection criteria, and findings), and specifics related to the implementation of interventions (such as technological platforms used, dissemination strategies, modes of usage, and developmental phase). Following the extraction of information from all included articles, descriptive quantitative analysis was used to summarize the frequency and distribution of native apps across platforms, study design, caregiver participant-selection criteria, and investigated outcomes. We conducted a comprehensive review of the included studies, focusing on specific aspects such as interventions and intervention outcomes. All relevant articles were thoroughly reviewed and coded to identify recurring patterns or concepts. These codes were grouped into categories based on similarities. Through several rounds of discussion and refinement, the authors identified major themes. The final themes were reached through consensus among the authors to ensure accuracy and consistency. For patients, caregivers, and telemedicine platforms, we summarized the key factors in telemedicine for patients with dementia.

## Results

### Selection and Inclusion of Studies

**Figure 1:**
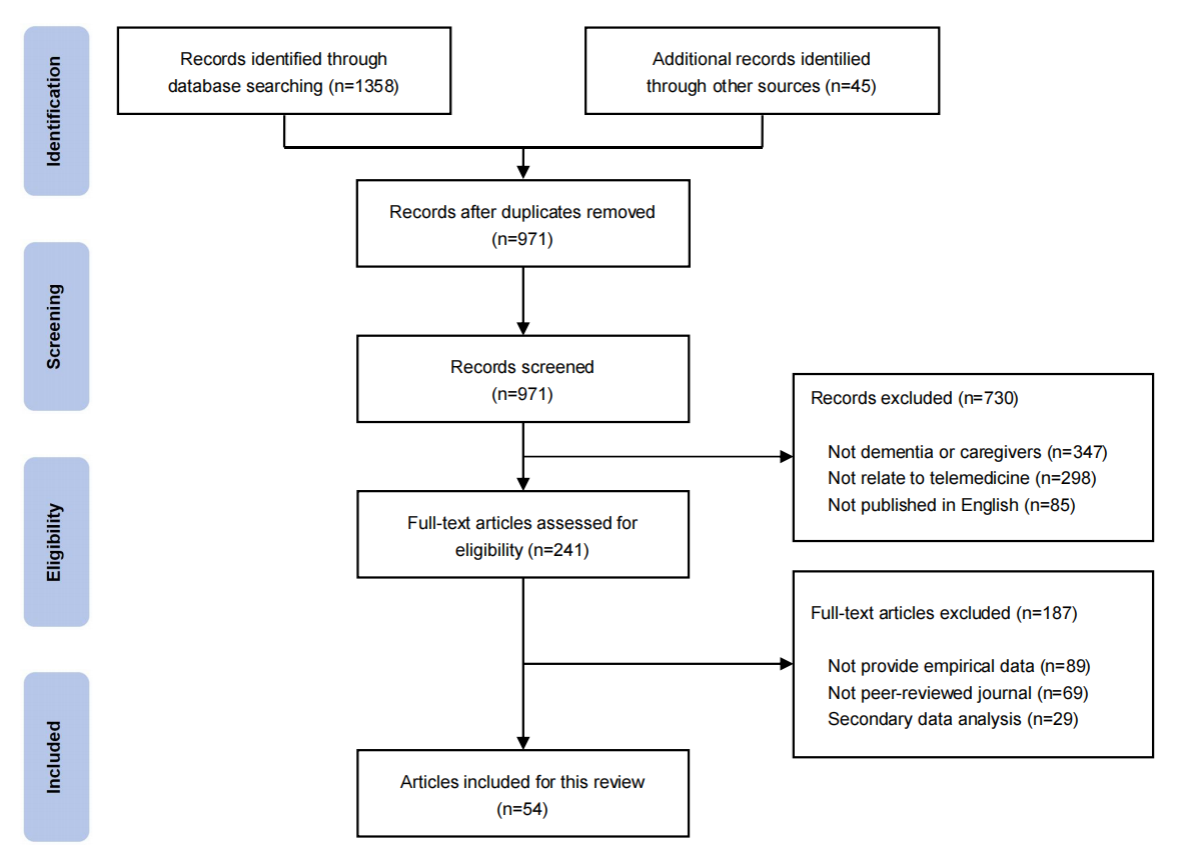
PRISMA (Preferred Reporting Items for Systematic Reviews and Meta-Analyses) flow diagram of the literature search.

A total of 1403 records were initially identified, with 1358 originating from the database search and 45 from other sources. After removing 432 duplicate records, a title and abstract screening process was conducted on the remaining 971 records. A total of 241 full-text articles were evaluated, and 730 articles were excluded from the study due to not having dementia or caregivers (n=347), not being related to telemedicine (n=298), and not being published in English (n=85). The final 54 articles were included in this study, while 187 articles were excluded due to not providing empirical data (n=89), not being peer-reviewed journals (n=69), secondary data analyses (n=29; see Figure 1 for details and Multimedia Appendix 1 for the PRISMA-ScR checklist).

### Study Characteristics

Tables 1 and 2 provide an overview of the included articles. These 54 studies were conducted across 5 different regions: 3 from Asia, 5 from Oceania, 24 from Europe, 20 from North America, 1 from South America, and 1 multinational study. In total, 27 articles were randomized controlled trials, 7 articles were single group intervention, and 9 articles reported qualitative research results. According to the study objective, 25 articles focused exclusively on dementia, and 9 articles focused exclusively on caregivers. Additional details regarding themes and related terms, search strategies for some of the databases searched, and the characteristics of the included studies are outlined in Multimedia Appendix 2.

**Table 1:** Information of the studies that were included.

Studies (author, year)	Country	Type of research^a^	Sample	Participants	Objects	Delivery method	Duration^b^ (months)
Lima et al, 2022 [15]	Brazil	SGI^c^	89	Mixed	Dementia	Mobile phone	8
Roach et al, 2021 [16]	Canada	Descriptive	20	Mixed	Mixed	Mobile phone	1
Lai et al, 2020 [17]	China	RCT^d^	60	Mixed	Mixed	Mixed	1
Arighi et al, 2021 [18]	Italy	RCT	108	Mixed	Mixed	Mixed	3
Capozzo et al, 2020 [19]	Italy	SGI	33	Mixed	Mixed	Mobile phone	1
Goodman-Casanova et al, 2020 [20]	Spain	RCT	93	Mixed	Mixed	Mobile phone	1
Marinello et al, 2021 [21]	Italy	Case study	10	Other dementia	Dementia	Mobile phone	3
Cheung and Peri, 2021 [22]	United Kingdom	Case study	10	Mixed	Dementia	Mixed	3
Cooper et al, 2021 [23]	United Kingdom	SGI	12	Mixed	Dementia	Mixed	2.5
Peri et al, 2023 [24]	New Zealand	Cross-sectional	38	Mixed	Mixed	Computer	3
Di Lorito et al, 2021 [25]	United Kingdom	Qualitative	10	Mixed	Mixed	Mobile phone	3
Giebel et al, 2021 [26]	United Kingdom	RCT	50	Mixed	Mixed	Mobile phone	1
Tuijt et al, 2021 [27]	United Kingdom	Qualitative	61	Mixed	Mixed	Mixed	3
Kalicki et al, 2021 [28]	United States	Cross-sectional	873	Mixed	Mixed	Mixed	2
Macchi et al, 2021 [29]	United States	Qualitative	198	AD^e^	Dementia	Mobile phone	4.5
Gately et al, 2022 [30]	United States	Cross-sectional	15	Other dementia	Dementia	Mixed	2
Masoud et al, 2021 [31]	United States	SGI	17	AD	Mixed	Computer	1
Weiss et al, 2021 [32]	United States	Descriptive	85	Other dementia	Caregivers	Mobile phone	2.5
Neal et al, 2023 [33]	Netherlands	RCT	150	Mixed	Mixed	Computer	30
Lott et al, 2006 [34]	United States	SGI	90	Alzheimer	Dementia	Mobile phone	2
Ganguli et al, 2023 [35]	United States	SGI	4691	Mixed	Dementia	Mixed	3.5
Gillespie et al, 2019 [36]	United States	RCT	731	Other dementia	Dementia	Mobile phone	5
Xie et al, 2018 [37]	United States	Cross-sectional	50	Other dementia	Mixed	Mixed	0.75
LaMonica et al, 2017 [38]	Australia	Descriptive	221	Other dementia	Dementia	Mixed	20
Cristancho-Lacroix et al, 2015 [39]	France	RCT	49	Alzheimer	Caregivers	Computer	6
Laver et al, 2020 [40]	Australia	RCT	104	Other dementia	Dementia	Mixed	4
Williams et al, 2019 [41]	United States	RCT	107	Other dementia	Dementia	Mobile phone	3
Howard et al, 2021 [42]	United Kingdom	Cross-sectional	495	Mixed	Dementia	Mixed	38
Wesselman et al, 2020 [43]	Netherlands	RCT	137	Mixed	Dementia	Mixed	1
Peterson et al, 2023 [44]	United States	Cross-sectional	27	Mixed	Mixed	Mixed	3
Iyer et al, 2023 [45]	United States	Qualitative	30	Mixed	Caregivers	Mobile phone	9
Lindauer et al, 2017 [46]	United States	RCT	28	Alzheimer	Mixed	Mixed	3
Mahoney et al, 2003 [47]	United States	RCT	100	Alzheimer	Caregivers	Mixed	12
Smith et al, 2023 [48]	United States	RCT	3838	Alzheimer	Dementia	Mixed	24
Emedoli et al, 2023 [49]	Italy	Cross-sectional	225	Mixed	Dementia	Computer	8
Carotenuto et al, 2018 [50]	Italy	RCT	28	Alzheimer	Dementia	Mixed	24
O'Connor et al, 2014 [51]	Australia	RCT	415	Alzheimer	Dementia	Mixed	4
Stara et al, 2021 [52]	Italy	Descriptive	20	Mixed	Mixed	Mixed	1
Mendez et al, 2021 [53]	United States	Cross-sectional	117	Mixed	Caregivers	Mixed	14
Lancaster et al, 2020 [54]	United Kingdom	Qualitative	35	Mixed	Dementia	Mobile phone	12
Potts et al, 2020 [55]	United Kingdom	RCT	56	Alzheimer	Mixed	Mixed	3
Banbury et al, 2019 [56]	Australia	RCT	69	Alzheimer	Dementia	Mixed	1.5
Williams et al, 2021 [57]	United Kingdom	RCT	71	Alzheimer	Mixed	Mixed	3
Pot et al, 2015 [58]	Netherlands	RCT	149	Alzheimer	Dementia	Computer	6
Pagán-Ortiz et al, 2014 [59]	United States	RCT	23	Alzheimer	Dementia	Mixed	12
Levinson et al, 2020 [60]	Canada	Cross-sectional	12	Alzheimer	Dementia	Mixed	5
Dam et al, 2019 [61]	Netherlands	RCT	96	Alzheimer	Caregivers	Mixed	4
Boots et al, 2017 [62]	Netherlands	RCT	72	Alzheimer	Mixed	Mixed	2
Baruah et al, 2020 [63]	India	RCT	23	Alzheimer	Caregivers	Computer	6
Mitchell et al, 2020 [64]	United States	RCT	30	Mixed	Mixed	Mixed	24
Núñez-Naveira et al, 2016 [65]	Spain	RCT	77	Alzheimer	Dementia	Mixed	3
Boessen et al, 2017 [66]	Netherlands	SGI	32	Alzheimer	Caregivers	Mixed	2.5
Park et al, 2020 [67]	Korea	RCT	26	Alzheimer	Dementia	Mobile phone	1
Gaugler et al, 2024 [68]	United States	RCT	240	Mixed	Caregivers	Mixed	12

^a^Descriptive refers to studies that describe characteristics or phenomena without exploring causal relationships or underlying mechanisms. Qualitative refers to studies that collect data through methods such as interviews or focus groups to explore participants' experiences, perspectives, or behaviors. Cross-sectional refers to studies that collect data at a single point in time, typically using surveys, to assess relationships or prevalence of variables.

^b^Data collection duration (months).

^c^SGI: single group intervention.

^d^RCT: randomized controlled trial.

^e^AD: Alzheimer disease.

**Table 2:** Characteristics of included studies.

			Studies (N=54)		Sample size (N=14,446)
**Regions, n (%)**					
	Asia	3 (5.56)		109 (0.75)	
	Oceania	5 (9.26)		847 (5.86)	
	Europe	24 (44.44)		2079 (14.39)	
	North America	20 (37.04)		11,295 (78.19)	
	South America	1 (1.85)		89 (0.62)	
	Multinational	1 (1.85)		27 (0.19)	
**Participants, n (%)**					
	Alzheimer	21 (38.89)		5469 (37.86)	
	Other dementia	8 (14.81)		1323 (9.16)	
	Mixed	25 (46.30)		7654 (52.98)	
**Research design, n (%)**					
	RCT^a^	27 (50.00)		6930 (47.97)	
	SGI^b^	7 (12.96)		4964 (34.36)	
	Case study	2 (3.70)		20 (0.14)	
	Cross-sectional	9 (16.67)		1852 (12.82)	
	Qualitative	5 (9.26)		334 (2.31)	
	Descriptive	4 (7.41)		346 (2.40)	
**Delivery method**					
	Mobile phone only	15 (27.78)		1607 (11.12)	
	Computer only	7 (12.96)		651 (4.51)	
	Mixed	32 (59.26)		12,188 (84.37)	
**Objects**					
	Dementia only	25 (46.30)		11,807 (81.73)	
	Caregivers only	9 (16.67)		772 (5.34)	
	Mixed	20 (37.04)		1867 (12.92)	
**Duration (months)**					
	<3	18 (33.33)		1754 (12.14)	
	3-12	26 (48.15)		7656 (53.00)	
	>12	10 (18.52)		5037 (34.87)	

^a^RCT: randomized controlled trial.

^b^SGI: single group intervention.

### Characteristics of the Study Design

Numerous researchers have worked to enhance telemedicine services by focusing on improving access and user-friendliness for individuals with dementia and their caregivers. Several reviewed studies established eligibility criteria to ensure that participants had access to the internet and were engaged with the service, with the goal of minimizing attrition rates. However, these criteria may unintentionally decrease the diversity and representation of the study sample by excluding individuals who may encounter significant barriers in using telemedicine technology. For instance, 2 articles excluded older adults lacking electronic devices or caregivers and those with physical disabilities, citing challenges in internet connectivity [15,17]. The complete list of included articles is provided in Table S3 in the Multimedia Appendix.

### Characteristics of Telemedicine Services

Various technological platforms, such as the web, telephone, mobile app, and remote wearable device, were used to administer interventions. The duration of telemedicine interventions varied, with some providing continuous access to websites, while others involved brief sessions, such as 6-minute telephone calls. Psychoeducation, psychotherapy, and social support were the most common types of interventions, even though the content varied. The psychotherapy interventions incorporated components of cognitive behavioral therapy aimed at assisting caregivers in regulating their emotions and behaviors, such as cognitive restructuring, relaxation techniques, and the use of telephone counseling. In total, 2 studies used the nation's predominant instant messaging application (ie, WhatsApp) for medical consultations [15,54]. Some programs developed user guides to assist older individuals with hearing impairments in navigating speakerphones. The assessment of outcomes varied among studies, with depression, anxiety, burden, and self-efficacy being the most frequently evaluated variables. Moreover, it was reported that interventions involving telephone, internet, and psychotherapy as well as social support had the larger effect sizes [28,55].

### Outcomes of People Living With Dementia

The term “dementia” broadly includes individuals with Alzheimer disease, vascular dementia, and early-onset dementia; nonetheless, 45.3% (24 out of 53) of the studies failed to specify the specific type of dementia. There was a significant correlation between characteristics and telemedicine service access among patients with dementia. A total of 3 studies used purposeful sampling techniques to enhance the diversity of participant representation, including individuals from racial and ethnic minority groups, individuals residing in rural areas, and families with varying income levels [25,33,35]. Elderly individuals with limited educational attainment displayed a decreased propensity to embrace telemedicine services.

### Outcomes of Caregivers

In total, 8 studies were developed to target caregivers, designed to improve their caregiving abilities, including the evaluation and management of symptoms, as well as offering professional consultation and psychological support. Various caregiver-focused interventions included eHealth, support group interventions, and care coordination. Nevertheless, the specific details regarding the execution of these interventions were still ambiguous. Formal caregivers typically possess greater expertise in providing care for individuals with dementia, as they have received specialized training and have accumulated prior experience in this area [69]. In addition, technology-based interventions may not be universally suitable for all caregivers due to various factors, such as age.

### Telemedicine Solutions and Mobile Device Platforms

**Figure 2:**
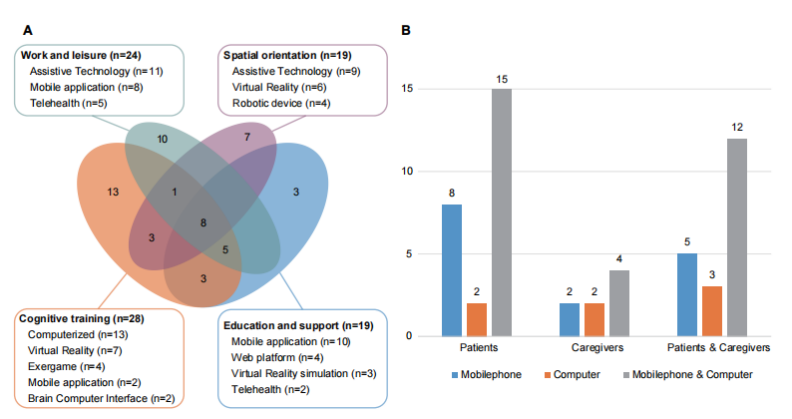
Telemedicine solutions and mobile device platforms. (A) Categorization of the telemedicine interventions by application domain. (B) Mobile device platforms used by patients and caregivers.

As shown in Figure 2A, the solutions could be categorized by application domain. Our sample included applications from all domains, but we found considerable overlap between the domains. Cognitive training was addressed in 28 studies. Within the domain of work and leisure, 24 solutions were identified. In addition to solutions for personal organization, these solutions also addressed the training and support for activities of daily living (eg, cooking, medication management, and household chores). There was another solution in this field that delivered education and support through telemedicine (n=19). The majority of mobility and navigation were focused on supporting spatial orientation and autonomous navigation (n=20). It was found that mobile phones were the most commonly examined mobile platforms, but preferences varied according to patients and caregivers (Figure 2A). Mobile devices are being investigated as convenient, cost-effective methods for screening and monitoring cognitive impairment due to the high rate of smartphone ownership. Several studies have developed ambulatory wearable sensors that use mobile phone to monitor voice, activity, and location.

### Effectiveness of Telemedicine

Overall, individuals with dementia and their caregivers reported satisfaction with telemedicine services and expressed willingness to continue using them. Panerai et al [59] conducted a study in Italy examining the effects of a telephone-based cognitive intervention on individuals living with dementia. The results indicated that the intervention group (n=14) demonstrated superior outcomes compared to the control group (n=13) after 1 month of training, including reductions in behavioral and psychiatric symptoms and improvements in cognitive performance. Capozzo et al [19] conducted a study in which they assessed the self-efficacy of caregivers and observed a notable enhancement following telemedicine. Additionally, several caregivers reported improved relationships with individuals living with dementia, highlighting the potential of telemedicine to foster emotional connections. In contrast, Goodman-Casanova et al [20] modified a television-based intervention for dementia by integrating COVID-19–related modules and health resources; however, their study found no statistically significant differences in outcomes, suggesting limited efficacy of this specific approach. There was no statistically significant difference observed in the outcomes of individuals living with dementia.

### Barriers in Accessing and Using Telemedicine Services

The challenges associated with implementing telemedicine for individuals with dementia and their caregivers can be categorized into 3 main domains: individual factors, accessibility-related factors, and environmental factors. Due to diminished cognitive abilities, individuals affected by dementia often encounter difficulties in concentrating and effectively communicating with health care professionals through digital platforms. Studies reported that individuals with advanced cognitive impairment, along with additional disabilities such as visual and auditory impairments, exhibit lower rates of engagement with telemedicine services compared to those in the early stages of dementia. In addition, the prevalence of the digital divide and inequality is significant among older adults. One study revealed that domestic broadband network coverage in households where individuals with dementia live alone was just 34%, which significantly limits their ability to access telemedicine services, particularly in underserved regions [18]. Several interventions and treatments, such as clinical tests necessitating specialized equipment and physical training and rehabilitation requiring close physical supervision by therapists, were not feasible to administer via telemedicine.

## Discussion

### Overview

Recent reimbursement policies have expanded access to telemedicine, making it an increasingly prominent method of remote care provision [70]. This study examined the feasibility, acceptability, and efficacy of telemedicine interventions for individuals with dementia and their caregivers. Overall, the results suggest that telemedicine programs hold promise in addressing the medical, psychological, and social needs and preferences of individuals in dementia-caregiver dyads.

### Telemedicine for Participants

This convenience is particularly beneficial for patients with dementia and their caregivers, who often face significant logistical challenges in accessing specialized health care. Participants in rural areas, who often face logistical barriers to accessing specialized care, perceive telemedicine to be as effective as in-person visits due to reduced commuting times [71]. This convenience is especially advantageous for patients with dementia and caregivers, facilitating timely access to health care. And it facilitates the involvement of family members who may be unable to physically attend a visit in person, allowing for greater support and collaboration in managing the patient's condition [72]. Telemedicine interventions, such as remote monitoring, digital consultations, and tele-education, have been shown to enhance medication adherence, reduce hospitalizations, and improve the overall quality of life for patients with dementia [20,35,42]. However, telemedicine implementation faces challenges when patients lack confidence or perceive a disconnect between the intervention’s goals and the organization’s mission [73].

### Telemedicine for Caregivers

According to a review, telemedicine is effective in addressing psychological concerns of caregivers of dementia [74]. However, the implementation of telemedicine is hindered by personal attributes of caregivers, such as deficiencies in their personal capacity (eg, financial and physical capacity, digital literacy), as well as staff members' lack of social and cultural awareness [75]. Therefore, the knowledge and capacity of caregivers are improved by using a cascade training model, hiring external training agencies, and requiring licensed and certified intervention caregivers to ensure program quality [76]. Caregivers must promptly address patient inquiries, adhere to telemedicine schedules, and conduct root-cause analyses to resolve recurring issues [77].

### Intervention Characteristics for Implementation

In the delivery method, video calls are determined to be more effective than phone calls, as a higher level of accuracy in their assessments is based on participants’ facial expressions and body language [48]. While telemedicine commonly uses video-based communication, instances of technical challenges or residents who may struggle with video interaction may require a transition to audio-based communication [78]. Although audio-based visits may present challenges for cognitive evaluations, there are telephone-based measures available that could provide valuable information to the specialist regarding the patient's condition [79]. It is imperative that providers receive supplementary similarly for all modalities of telemedicine. In order to compare interventions effectively, rigorous and standardized methods are required [80].

### Outer Environmental Setting

External factors, such as insurance coverage, policy regulations, and broadband accessibility, significantly influence telemedicine implementation. However, these aspects were underexplored, with most studies focusing on barriers like reimbursement policies and digital infrastructure gaps [81]. For example, support from external sources assists in circumventing additional implementation costs. Telemedicine methods are more likely to be covered by insurers for health care encounters of patients with dementia [42]. To enhance the effectiveness of telemedicine, future studies should examine its integration with local health systems, addressing specific needs of patients, caregivers, and communities. Practical steps, such as training local staff and tailoring interventions to cultural contexts, can further improve outcomes [82].

### Factors to Consider for Successful Implementation of Telemedicine

**Figure 3:**
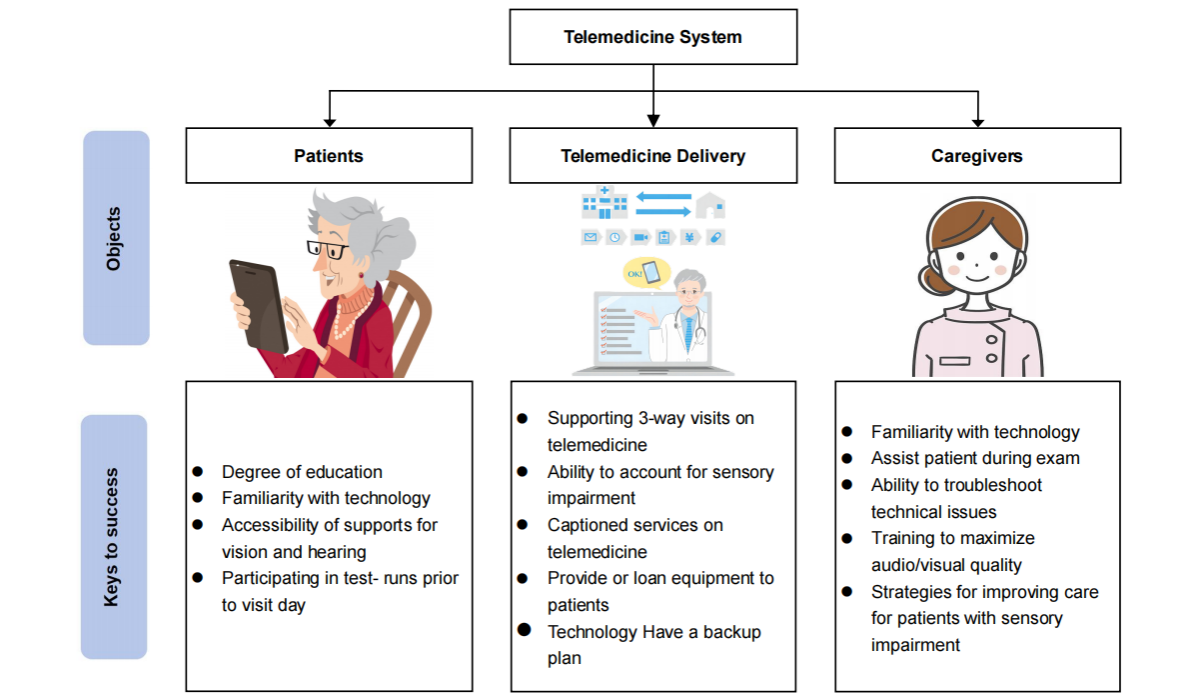
Factors to consider for successful implementation of telemedicine for patients with dementia.

In order to successfully implement telemedicine systems for patients with dementia, it is necessary to take into account both their needs and the way they interact with telemedicine (Figure 3). Adapting technology that accounts for sensory impairments is one possible way to accommodate the needs of patients with dementia [83]. Considering the high prevalence of hearing loss among up to 90% of older adults with Alzheimer disease and related dementias, as well as the substantial impact of vision impairment affecting over 30% of individuals with Alzheimer disease and related dementias, the modification of telemedicine technologies to accommodate sensory changes holds promise for enhancing the efficacy of telemedicine services for this population [40]. Additionally, it may be advantageous for caregivers to receive specialized training in effectively communicating with patients with dementia who experience cognitive and sensory impairments [84]. Interdisciplinary care team members can use this information to contact patients ahead of time about specific barriers to care. Depending on the patient's specific needs, a patient-specific plan might require training care partners in telemedicine, equipment, or captioning services, as depicted in Figure 3.

### Why Is Telemedicine Effective?

First, telemedicine can make people with dementia feel more comfortable, reduce their anxiety about unfamiliar environments, and enhance the effectiveness of interventions or treatments [44]. Second, videoconferencing platforms provide therapists with the ability to use visual and auditory stimuli to engage individuals with dementia, thereby enhancing communication [23]. Third, caregivers have reported that telemedicine services help to reduce the burden of travel time and financial expenses associated with visiting clinics or treatment centers [85].

### Barriers in Telemedicine Services

The field of telemedicine research encounters numerous obstacles, including the provision of access to telemedicine services for individuals who have limited experience with remote or internet-based platforms, as well as the verification of the efficacy and effectiveness of these services [86]. Patients with dementia may face barriers in accessing and using technology due to the digital divide. For instance, the rate of smartphone ownership among individuals aged 65 years and older is 61%, the figure is notably lower than the 90% ownership rate observed among individuals aged 18-35 years [87]. Besides, some participants raised concerns regarding safety and privacy when using telemedicine services [88]. However, existing studies have not documented the implementation of adequate cybersecurity measures to safeguard personal health information. Finally, most of the studies are in the pilot phase of development, which raises questions about their effectiveness [89].

### Work in the Future

Based on the results of this review, there are numerous potential avenues for further research. First, additional research is required to create implementation frameworks for telemedicine that effectively guide the implementation processes and overcome barriers in community-based implementation of non-pharmacological evidence-based interventions [90]. Second, the optimization and enhancement of telemedicine programs is essential for ongoing improvement. For instance, the use of a wearable device to track a patient's heart rate and blood pressure in the context of a telemedicine consultation can improve the evaluation of the individual's health status, thereby contributing to enhanced patient care [91]. Third, telemedicine services should explore the incorporation of emotional support modules, including emotional health screening, psychological counseling, and referrals to mental health services. In order to bridge the digital divide in the future, more investment and technical guidance will be needed [92].

Finally, future research should focus on implementing more robust data encryption techniques in telemedicine platforms, particularly during data transmission and storage. Research could explore the effectiveness of various combinations, such as passwords, message verification codes, and biometric authentication, in enhancing system security [93]. In addition, educating patients and caregivers on cybersecurity practices is essential. For example, educational initiatives on identifying phishing attacks, using strong passwords, and configuring secure settings would be valuable [94]. Furthermore, developing industry standards and compliance guidelines would provide a framework for data security in telemedicine, ensuring that patient health information remains protected in line with global privacy regulations [95].

### Limitations

Our study also has limitations that warrant discussion. First, the implementation of telemedicine relies on the familiarity of patients and caregivers with technology. Many patients and caregivers may lack the technical skills to use devices such as smartphones and computers, which could prevent some individuals from effectively participating in telemedicine services, thereby limiting the generalizability of the study findings. Second, most studies focus on the short-term effects of interventions, with a lack of evaluation of the long-term impact of telemedicine. Dementia is a chronic progressive disease, and short-term outcomes may not fully reflect the actual role of telemedicine in long-term care. Finally, the telemedicine interventions included in studies are highly diverse, with no standardized protocols or evaluation criteria. This variability makes direct comparisons between studies difficult, affecting the reliability and generalizability of research conclusions.

### Conclusions

The study reviewed indicates that telemedicine services show promise in addressing the difficulties encountered by individuals with dementia and their caregivers, including enhancing mental well-being, preserving quality of life, and averting functional deterioration. Collaborative efforts at multiple levels are necessary to address the digital divide in order to enhance their access to telemedicine programs and improve readiness for potential future emergencies. Future research should consider a dyadic perspective to enhance comprehension of the requirements of individuals with dementia and their caregivers, thereby improving the design and efficacy of telemedicine programs.

## Data Availability

All data generated or analyzed during this study are included in this published article and its supplementary information files.

## Appendix

Multimedia Appendix 1 PRISMA-ScR (Preferred Reporting Items for Systematic Reviews and Meta-Analyses extension for Scoping Reviews) checklist.

Multimedia Appendix 2 Additional information.

## Abbreviations

CONSORT-EHEALTH: Consolidated Standards of Reporting Trials of Electronic and Mobile Health Applications and Online Telehealth
MeSH: Medical Subject Headings
PRISMA-ScR: Preferred Reporting Items for Systematic Reviews and Meta-Analyses extension for Scoping Reviews
