# Supplementary material for: Implementation of Telemedicine for Patients With Dementia and Their Caregivers: Scoping Review
Source: J Med Internet Res. 2025 May 5;27:e65667. doi: 10.2196/65667 (PMC12089872; doi:10.2196/65667)
Supplement: Multimedia Appendix 2 [file jmir_v27i1e65667_app2.docx]

**Supplementary information**

[Table 1. The themes and the related terms 1](#_Toc23496)

[Table 2. Search strategies for some databases searched 2](#_Toc14463)

[Table 3. Characteristics of included studies 4](#_Toc27088)

### Table 1. The themes and the related terms

| **Themes** | **Related terms** |
| --- | --- |
| Dementia | Dementia  Dementias  Alzheimer-Type  Alzheimer's  Alzheimer  Vascular dementia  Cognition impairment  Frontotemporal lobar degeneration  Lewy body disease  Huntington’s disease |
| Telemedicine | Digitization  Telemanagement  Tele management  remote management  remote administration  tele medicine  telemedicine  Tele-Referral  Tele Referral  Tele-Referrals  Telehealth  eHealth  Telecare  Tele-Care  TeleCare  Tele-Intensive Care  Tele Intensive Care  Mobile Health  Health, Mobile  mHealth |

### Table 2. Search strategies for some databases searched

| **Pubmed** |
| --- |
| (((((((((Dementia[Title/Abstract]) OR (Dementias[Title/Abstract])) OR (Alzheimer-Type[Title/Abstract])) OR (Alzheimer's[Title/Abstract])) OR (Alzheimer[Title/Abstract])) OR (Vascular dementia[Title/Abstract])) OR (Cognition impairment[Title/Abstract])) OR (Frontotemporal lobar degeneration[Title/Abstract])) OR (Lewy body disease[Title/Abstract])) OR (Huntington’s disease[Title/Abstract]) AND (((((((((((((((((((Digitization[Title/Abstract]) OR (Telemanagement[Title/Abstract])) OR (Tele management[Title/Abstract])) OR (remote management[Title/Abstract])) OR (remote administration[Title/Abstract])) OR (tele medicine[Title/Abstract])) OR (telemedicine[Title/Abstract])) OR (Tele-Referral[Title/Abstract])) OR (Tele Referral[Title/Abstract])) OR (Tele-Referrals[Title/Abstract])) OR (Telehealth[Title/Abstract])) OR (eHealth[Title/Abstract])) OR (Telecare[Title/Abstract])) OR (Tele-Care[Title/Abstract])) OR (Tele Care[Title/Abstract])) OR (Tele-Intensive Care[Title/Abstract])) OR (Tele Intensive Care[Title/Abstract])) OR (Mobile Health[Title/Abstract])) OR (Health, Mobile[Title/Abstract])) OR (mHealth[Title/Abstract]) |
| **C****ochrane Library** |
| ((Dementia[Title/Abstract]):ti,ab,kw OR (Dementias[Title/Abstract]):ti,ab,kw OR (Alzheimer-Type[Title/Abstract]):ti,ab,kw OR (Alzheimer's[Title/Abstract]):ti,ab,kw OR (Alzheimer[Title/Abstract]):ti,ab,kw OR (Vascular dementia[Title/Abstract]):ti,ab,kw OR (Cognition impairment[Title/Abstract]):ti,ab,kw OR (Frontotemporal lobar degeneration[Title/Abstract]):ti,ab,kw OR (Lewy body disease[Title/Abstract]):ti,ab,kw OR (Huntington’s disease[Title/Abstract]):ti,ab,kw) AND ((Digitization[Title/Abstract]):ti,ab,kw OR (Telemanagement[Title/Abstract]):ti,ab,kw OR (Tele management[Title/Abstract]):ti,ab,kw OR (remote management[Title/Abstract]):ti,ab,kw OR (remote administration[Title/Abstract]):ti,ab,kw OR (tele medicine[Title/Abstract]):ti,ab,kw OR (telemedicine[Title/Abstract]):ti,ab,kw OR (Tele-Referral[Title/Abstract]):ti,ab,kw OR (Tele Referral[Title/Abstract]):ti,ab,kw OR (Tele-Referrals[Title/Abstract]):ti,ab,kw OR (Telehealth[Title/Abstract]):ti,ab,kw OR (eHealth[Title/Abstract]):ti,ab,kw OR (Telecare[Title/Abstract]):ti,ab,kw OR (Tele-Care[Title/Abstract]):ti,ab,kw OR (Tele Care[Title/Abstract]):ti,ab,kw OR (Tele-Intensive Care[Title/Abstract]):ti,ab,kw OR (Tele Intensive Care[Title/Abstract]):ti,ab,kw OR (Mobile Health[Title/Abstract]):ti,ab,kw OR (Health, Mobile[Title/Abstract]):ti,ab,kw OR (mHealth[Title/Abstract]):ti,ab,kw) |
| **PsycINFO** |
| ('Dementia':ad,ti OR 'Dementias':ad,ti OR 'Alzheimer-Type':ad,ti OR 'Alzheimer's':ad,ti OR 'Alzheimer':ad,ti OR 'Vascular dementia':ad,ti OR 'Cognition impairment':ad,ti OR 'Frontotemporal lobar degeneration':ad,ti OR 'Lewy body disease':ad,ti OR 'Huntington’s disease':ad,ti) AND ('Digitization':ad,ti OR 'Telemanagement':ad,ti OR 'Tele management':ad,ti OR 'remote management':ad,ti OR 'remote administration':ad,ti OR 'tele medicine':ad,ti OR 'telemedicine':ad,ti OR 'Tele-Referral':ad,ti OR 'Tele Referral':ad,ti OR 'Tele-Referrals':ad,ti OR 'Telehealth':ad,ti OR 'eHealth':ad,ti OR 'Telecare':ad,ti OR 'Tele-Care':ad,ti OR 'Tele Care':ad,ti OR 'Tele-Intensive Care':ad,ti OR 'Tele Intensive Care':ad,ti OR 'Mobile Health':ad,ti OR 'Health, Mobile':ad,ti OR 'mHealth':ad,ti) |
| **EMBASE** |
| ('Dementia':ad,ti OR 'Dementias':ad,ti OR 'Alzheimer-Type':ad,ti OR 'Alzheimer's':ad,ti OR 'Alzheimer':ad,ti OR 'Vascular dementia':ad,ti OR 'Cognition impairment':ad,ti OR 'Frontotemporal lobar degeneration':ad,ti OR 'Lewy body disease':ad,ti OR 'Huntington’s disease':ad,ti) AND ('Digitization':ad,ti OR 'Telemanagement':ad,ti OR 'Tele management':ad,ti OR 'remote management':ad,ti OR 'remote administration':ad,ti OR 'tele medicine':ad,ti OR 'telemedicine':ad,ti OR 'Tele-Referral':ad,ti OR 'Tele Referral':ad,ti OR 'Tele-Referrals':ad,ti OR 'Telehealth':ad,ti OR 'eHealth':ad,ti OR 'Telecare':ad,ti OR 'Tele-Care':ad,ti OR 'Tele Care':ad,ti OR 'Tele-Intensive Care':ad,ti OR 'Tele Intensive Care':ad,ti OR 'Mobile Health':ad,ti OR 'Health, Mobile':ad,ti OR 'mHealth':ad,ti) |

### Table 3. Characteristics of included studies

| **References (author, year)** | **Categorization of Themes** | **Outcome measures** | **Service** | **Major Findings** |
| --- | --- | --- | --- | --- |
| Lima et al., 2022 | To describe the feasibility of Telemedicine tools, including patient recruitment, attendance, discomfort, satisfaction, and travel time/cost savings. | Assessment Staging Tool; the Katz Activities of Daily Living scale; and the Abbreviated Mental Test 4. | WhatsApp-based remote medical consultation for people living with dementia. Intervention includes text and video treatment, health promotion, drug prescription, diagnostic tests, and referrals. | Recruitment rate: 85.5% (89/104); Attendance rate: 97.7% (87/89); Completion rate: 97.7% (85/87); Discomfort rate: 9.4% (8/85); Satisfaction rate: 90% accept the telemedicine approach, 45% believe it's effective; Saved 233 min and 11 USD on travel each time. |
| Roach et al., 2021 | Reflexive thematic analysis is used to analyze the interview and field note data. | Folstein Mini-Mental Status Examination | The presence of a clinical research nurse on the team, familiar with the clinic population, meant that only people with dementia with the capacity to consent and participate if they wished to were approached to consent to the study. Due to the nature of some of the clinic populations, explicit oral consent was necessary so that participants who wanted to participate but perhaps did not have access to the internet, e-mail, or who had disablements preventing them from using devices or computer screens could still be included. Explicit oral consent provided a way to ethically include a diverse population. | A reflexive thematic analysis was used to analyze the interview and field note data. The impacts of the public health measures in response to the pandemic emerged through iterative analysis in three main categories of experience:(1) personal, (2) health services, and (3) health status (of both persons living with dementia and care partner). |
| Lai et al., 2020 | To community services for older adults with neurocognitive disorder and their caregivers. | versions of the Montreal Cognitive Assessment, the Revised Memory and Behavior Problem Checklist, the Quality of Life in Alzheimer's Disease assessment, The Short Form 36 version 2, The Zarit Burden Interview Scale, and the Revised Caregiving Self Efficacy Scale. | The impact of additional services delivered to both care recipient and caregiver through video conference (n = 30) was compared with telehealth targeted at caregivers by telephone only (n = 30), over 4 weeks in a pretest−post-test design. Interviews and questionnaires were conducted at baseline and the study's end. | Telemedicine by video conference was associated with improved resilience and well-being for both people with NCD and their caregivers at home. |
| Arighi et al., 2021 | To describe the digital divide of a population of patients with dementia contacted by Telemedicine. | Mini-Mental State ExaminationIn | The software enabled neurologists to communicate with patients from their workstations, starting a video call, and using a headset. During the television, neurologists collected patients' sociodemographic information and the characteristics of the caregiver who assisted the patient. After that, the neurologist collected recent medical history, performed a brief neurological examination according to telehealth and remote care advice from the American Academy of Neurology, and either provided advice and prescriptions or modified the therapy. | Results Seventy-four patients connected with a neurologist (successful television, 68.5%), and 34 patients were not able to perform television and were contacted by phone (failed television, 31.5%). No significant differences were observed among the two groups concerning age, gender, and education, but the prevalence of successful television was higher in the presence of younger caregivers: televisions performed in the presence of subjects of younger generation (sons and grandsons) had a successful rate higher (86% successful, 14% failed) than the group without younger generation caregiver (49% successful, 51% failed). |
| Capozzo et al., 2020 | To evaluate multidisciplinary assessment of patients with dementia using telehealth during the COVID-19 pandemic. | A structured questionnaire and Clinical Dementia Rating Scale | The telemedicine assessment in this study was based on a structured questionnaire designed to collect demographics and clinical information [diagnosis, onset of disease, clinical status, and changes since last visit in the following domains: cognition, behavior and personality, language, sleep disturbances, nutritional status, swallowing capacity, respiratory function, access to rehabilitation and speech therapy, pharmacological therapy] to identify significant changes or problems since the last multidisciplinary evaluation (see Supplementary Material). | The study was conducted in Italy, one of the countries hit particularly hard by the COVID-19 pandemic, with the interruption of all non-emergency medical services. Our study indicates that telemedicine is a valid tool to triage patients with FTD to increase practice outreach and efficiency |
| Goodman-Casanova et al., 2020 | To provide television-based and telephone-based health and social support, and to study the effects of a television-based assistive integrated technology. | Overall well-being, Sleep quality | Telephone-based survey research was conducted according to Gordon's Functional Health Patterns. Overall, quantitative strategies (questions with numerically rated items) were used for data collection of the health perception management and sleep-rest patterns. Qualitative strategies (open-ended questions) were used for the coping-stress tolerance, activity-exercise, and role-relationship patterns. Data were organized into previously coded and listed categories using a directed content analysis approach. | Our findings suggest that during COVID-19 confinement, the physical and mental health and well-being were optimal for the majority of our vulnerable population. However, those living alone reported greater negative psychological effects and sleeping problems. |
| Marinello et al., 2021 | The hybrid in-person and tele-visit approach of assisted HaH could help to create a “secure” environment, empowering caregivers to manage frail older adults with COVID-19 at home. | We report one such atypical COVID-19 case that we were able to successfully manage at home thanks to her highly motivated caregivers and the support of a telemedicine solution (TMS). | Aspect and use of the telemedicine system. A shows Hospital at Home nursing and medical personnel wearing personal protective equipment while providing hospital-level care, including fluid infusion, to the patient. In the background, the remote station features an operator-controlled high-definition camera with built-in optical and digital zoom (arrowhead), a touchscreen monitor (asterisk), and a speakerphone (arrow). B shows Hospital at Home personnel using the control station to monitor the patient's condition communicating with caregivers through a high-fidelity monitor (asterisk), webcam (arrowhead), and speakerphone (arrow). Televisions are prompted by the control station and accepted by the remote station to ensure privacy. The control station allows to control of multiple televisions simultaneously | The hybrid in-person and tele-visit approach of TMS-assisted HaH could help to create a "secure" environment by addressing self-confidence and safety issues, thus empowering motivated caregivers to manage frail older adults with COVID-19 at home, avoiding unnecessary admissions to closed wards and their negative physical, functional, and psychological outcomes. |
| Cheung and Peri, 2021 | To actively engage and stimulate people with dementia. | CST has been shown to improve cognition, quality of life, and communication; and is recommended for people with mild to moderate dementia in the UK's NICE dementia guidelines | The Zoom video conferencing platform was used for our two to three weekly COP online forums, which served many purposes and generated several activities among the CST facilitators, including designing a pathway to implement CST, peer-reviewing the content of CST, sharing learning from facilitating CST, problem-solving challenges, and sharing CST resources. | Our case example illustrated how COVID-19 has fast-tracked the adoption of technology in dementia practice, which is likely to stay beyond COVID-19. Technology has many potentials to enhance the holistic management of dementia across the spectrum from prevention, risk reduction, early detection, diagnosis, assessment, care delivery, and living well with dementia (Astell et al., 2019). |
| Cooper et al., 2021 | To investigate the acceptability and feasibility of Telemedicine. | subjective cognitive impairment (SCD); The Functional Assessment Questionnaire scale | We developed a remote version that was similar in content and intended mechanisms of action to the planned face-to-face format, for delivery on Zoom. We added facilitator prompts acknowledging that lifestyle change may be more challenging and need adapting, in the pandemic context. Additionally, the participants were encouraged to continue to use three of the usual functionalities of TV-AssistDem which are meant to guarantee physical and mental health and well-being. Physical activity was promoted through the visualization of videos of indoor home exercise. Cognition was stimulated with Stimulus memory games. Lastly, social connectedness with loved ones and health professionals was facilitated through video calls | For more vulnerable populations, messages that lifestyle change can help memory should be communicated alongside supportive, relational approaches to enabling lifestyle changes. |
| Peri et al., 2023 | To explore the roles and experiences of carers in accessing virtual Cognitive Stimulation Therapy. | Using keywords from the interview questionnaire as a basic unit of analysis, the transcript data was coded accordingly using NVivo 12 and following the phases outlined by Elo and Kyngäs (2008). After this initial familiarization and categorization of the data content, both KP and DB crafted further coding in the search for themes or patterns across the data set. | Further review and defining of the theme/s occurred during subsequent meetings following the six-phase process: (1) familiarizing with the data; (2) generating initial codes; (3) searching for themes; (4) reviewing themes; (5) defining and naming themes; and (6) producing the report. | Accessing vCST provided carers with an immediate application of new knowledge. Carers reported positive responses to vCST that provided their family member living with dementia with social contact and cognitive stimulation during lockdown. |
| Di Lorito et al., 2021 | The Promoting Activity, Independence, and Stability in Early Dementia (PrAISED) is delivering an exercise program for people with dementia. | The participants with dementia and their caregivers were invited by the main researcher to take part in two semi-structured qualitative interviews. | Based on a topic guide developed in collaboration with two Patient and Public Involvement contributors with experience in caring for someone with dementia, who were also involved in the development of the study and its protocol, and the writing of this manuscript. While the topic guide focused on the overall experience of the PrAISED RCT, a flexible approach was used in the interviews to explore and capture information relating to Q Health. All the interviews were carried out through speakerphone so that participants and caregivers could both hear and respond to the questions. | Video delivery worked best when participants had a supporting caregiver and when therapists showed enthusiasm and had an established rapport with the client. Benefits included time efficiency of sessions, enhancing participants' motivation, caregivers' dementia awareness, and therapists' creativity. |
| Giebel et al., 2021 | To explore the effects of telemedicine-related social care and support service changes on the lives of unpaid carers. | Paired samples t-tests were used to compare the mean of weekly hours of social support service usage before and since the outbreak. | Thematic analysis identified three overarching themes: (1) Loss of control; (2) Uncertainty; and (3) Adapting and having to adapt to the new normal. | PLWD and carers need to receive specific practical and psychological support during the pandemic to support their well-being, which is severely affected by public health restrictions. |
| Tuijt et al., 2021 | To understand the remote healthcare experiences of patients living with dementia and their family carers. | Two authors individually analyzed COVID-19 data from the first 20 interviews and independently developed thematic frameworks. Frameworks had many coding similarities, and different codes and themes were discussed and refined to form a single framework, driven by the data. | Proactive care at the onset of COVID-19 restrictions; • avoidance of healthcare settings and services; and • difficulties with remote healthcare encounters | Three main themes were derived relating to proactive care at the onset of COVID-19 restrictions; avoidance of healthcare settings and services; and difficulties with remote healthcare encounters. People living with dementia and their carers felt check-up calls were reassuring but limited in scope and content. Some avoided healthcare services, wishing to minimize COVID-19 risk reduce NHS burden, or encounter technological barriers. Difficulties in remote consultations included a lack of prompts to remember problems, dealing with new emerging difficulties, rescheduling/missed calls, and inclusion of the voice of the person with dementia. |
| Kalicki et al., 2021 | To identify major barriers to video-based telehealth use among homebound older adults. | An 11-item questionnaire was developed to identify patients to supply with a video-based telehealth device. We identified multiple barriers to usage including lack of caregivers and lack of access to appropriate technology. | Physicians were asked to elaborate on barriers to access among their patient population, with questions regarding internet connectivity, ability to pay for data plans, and access to a computer or video-enabled device | According to physicians in the program, more than one-third (35%) of homebound patients (mean age of 82.7; 46.6% with dementia; mean of 4 comorbidities/patient) engaged in first-time video-based telehealth encounters between April and June 2020 during the first COVID-19 surge in NYC. The majority (82%) required assistance from a family member and/or paid caregiver to complete the visit. Among patients who had not used telehealth, providers deemed 27% (n = 153) "unable to interact over video" for reasons including cognitive or sensory impairment and 14% lacked access to a caregiver to assist them with technology. Physicians were not knowledgeable of their patients' internet connectivity, ability to pay for cellular plans, or video-capable device access. |
| Macchi et al., 2021 | To describe the impact of Telemedicine on patients living with chronic, neurodegenerative disease and their caregivers. | Semi-structured interviews, open-ended survey responses, medical record documentation, and participant-researcher communications | Interviews were conducted by teleconference or telephone using an iterative interview guide per the parent trial protocol. | While telemedicine has helped improve access to healthcare, patients and caregivers perceive clear limitations compared to in-person services. Changes in society and healthcare delivery in response to COVID-19 highlight ongoing and novel gaps that must be addressed to optimize future outpatient palliative care for neurologic illness. |
| Gately et al., 2022 | To increase connectedness between Veterans and patient-aligned care teams and improve quality of care through patient narratives developed using a guided interview process. | Mini-Mental State Examination(MMSE); Questionnaires included statements with five-point Likert scales of agreement ranging; a five-item questionnaire modified from a dementia telehealth study (Moo et al., 2014);5-point Likert scales. | Complete MLMS interviews with occupational therapy trainees using telehealth technology. | Veterans with cognitive concerns successfully participated in virtual MLMS interviews during COVID-19. Caregivers enhanced Veteran engagement and often provided technological support. |
| Masoud et al., 2021 | To provide individuals living with Dementia and care, partners, an opportunity to socialize in an inclusive and supportive environment. | A thorough review of existing literature about Memory Cafés was conducted to inform the development of the field notes and interview guides. | Memory Cafés, sometimes referred to as Alzheimer's or Dementia Cafés is a widely implemented program that provides individuals living with dementia and their care partners an opportunity to socialize with others | Memory Cafés offer important benefits for families living with dementia, providing vital new insight into the potential for virtual Memory Cafés to offer similar benefits. |
| Weiss et al., 2021 | To demonstrate a unique approach to reaching culturally diverse and vulnerable populations using telehealth, and share some of the lessons learned as a result of early difficulties. | depression/anxiety assessment tools | Television model: introduction, review of health records, treatment, caregiver stress evaluation, advance care planning, emergency planning, respite care, | Factors related to no-show: female, Black, high school graduates; Most feel positive and helpful; caregiver feel all concerns addressed; many hope to return to in-person but maintain television for follow-up; Can conduct real-time and accurate assessment; More inclusive and accessible. |
| Neal et al., 2023 | To evaluate the benefits of the FindMyApps intervention to people with mild dementia and their informal caregivers. | Adult Social Care Outcomes Toolkit, Maastricht Social Participation Profile, Short Sense of Competence Questionnaire, Dementia Quality of Life Instrument | Help people (learn to) use a tablet and find apps, that are user-friendly for people with mild dementia/MCI, which may facilitate social contact (e.g.through video calls, instant messaging, or multiplayer games) or self-management (e.g. medication reminder apps and diaries), and which meet an individual's needs and interests. | Caregivers who received FindMyApps had a moderately higher sense of competence at three months than those receiving digital care-as-usual. Investigation of effect modifiers suggested a more beneficial effect of Find MyApps on neuropsychiatric symptoms and engagement in pleasurable activities for people with a diagnosis of MCI, and for people with mild dementia/MCI with apathy at baseline on quality of life outcomes. |
| Lott et al., 2006 | To determine the feasibility of diagnosing dementia in remote populations of individuals and to compare aspects of the diagnostic process with those used in a traditional face-to-face academic medical-center based clinic site. | Dementia Questionnaire for Mentally Retarded Persons | Telemedicine has been defined as the use of electronic information and communication technologies to provide patient care when distance separates the participants。 | This study supports the need for formal reliability and validity studies of TM preparatory to the consideration of this modality for use in clinical trials for AD. |
| Ganguli et al., 2023 | To evaluate characteristics of Medicare beneficiaries associated with practices and clinicians offering telephone visits only and patients receiving telephone visits only, when both telephone and video were available. | Clinical and  technology variables | Telephone and video visits | This study found that many patients reported choosing telephone visits when given the option, suggesting the need to support telephone visits when appropriate while addressing multilevel barriers to video use (eg, clinic infrastructure and interpreter availability |
| Gillespie et al., 2019 | Access to Telemedicine services for older adults with dementia residing in senior living communities can effectively decrease Emergency Department utilization. | Emergency Department visits over time | When an intervention subject needed care and requested assistance from the geriatrics practice, the provider managed the issue via phone, an outpatient or Emergency Department visit, or a telemedicine visit | Telemedicine can effectively decrease Emergency Department use by individuals with dementia, but further research is needed to confirm this secondary analysis and to understand how to best implement and optimize telemedicine for patients with dementia suffering from acute illnesses. |
| Xie et al., 2018 | To examine caregivers' mobile device usage and their desire to receive information via mobile devices. | Instrumental Activities of Daily Living, Health Information Wants Questionnaire, Information Preference Scale, Decision-making Preference Scale. | To support health education or self-management for a wide range of health conditions, such as eating disorders, multiple sclerosis, cardiovascular disease, HIV, and mental illnesses, to name just a few. | There is much need for effective mHealth interventions that can provide information tailored to the needs and preferences of these caregivers. |
| Lamonica et al., 2017 | To describe patterns of Internet use, as well as interest in and preferences for eHealth technologies among older adults with varying degrees of cognitive impairment. | eHealth Questionnaire. | offer self-help through Web-based interventions, or deliver proactive and guided interventions. | Our data demonstrate an overwhelming interest within this demographic for targeted interventions to address modifiable risk factors for cognitive decline, particularly memory and computer-based exercises for cognition. These findings support future research efforts into the development, implementation, feasibility, and acceptability of eHealth interventions to support the health and well-being of individuals with cognitive impairment and their carers. As part of this process, it will be important to develop strategies to promote the use of eHealth technologies, including social media websites and apps among older adults with lower levels of education. |
| Cristancho-Lacroix et al., 2015 | To evaluate trial the efficacy and acceptability of a Web-based psychoeducational program for informal caregivers of persons with Alzheimer's disease based on a mixed methods research design. | Satisfaction questionnaire; standardized questionnaires, and visual analog scales. | Adapted and developed the Diapason program, based on a user-centered design, including a proof of concept and 2 usability tests. | They preferred the topics offering strategies to maintain the PWAD's autonomy and teaching skills for coping with behavioral problems but were less interested in self-care. |
| Laver et al., 2020 | To determine whether delivery of a dyadic intervention using telehealth was non-inferior to delivery of the same program using traditional face-to-face delivery through home visits. | Caregiving Mastery Index. | This may be used to complement other forms of communication such as e-mail and telephone use. Studies suggest that more interactive modalities (such as the Internet) are preferred to those that involve the use of the telephone alone | This study suggests that evidence-based dyadic interventions can be adapted for telehealth delivery and could be made available to those who are currently unable to access such interventions through home visits. |
| Williams et al., 2019 | To evaluate the effects of a telehealth intervention on caregiver outcomes. | Functional Assessment Scale. | Provide tailored dementia-care strategies to in-home caregivers based on video recordings caregivers submit of challenging care situations. | This research demonstrated the benefits of using available technology to link families to dementia care experts using video-recording technology |
| Howard et al., 2021 | The use of assistive technology and telecare has been promoted to manage risks associated with independent living in people with dementia. | BristolActivities of Daily Living Scale, Mini-Mental State Examination. | Assistive technology (AT) refers to electronic or mechanical devices that can support independence and improve quality of life by assisting with daily living activities, reducing harmful risks, and improving communication. Devices used in dementia care can be broadly categorized as reminder or prompting devices, monitors, and detectors to support safety, safer walking technologies, and communication devices and devices to support the use of leisure activities [7]. Telecare uses a combination of monitored alarms, sensors, and other equipment to help people live independently | Time living independently outside a care home was not signiﬁcantly longer in participants who received full ATT and ATT was not cost-eﬀective. Participants with full ATT attained fewer QALYs based on participant-reported EQ-5Dthan controls at 104 weeks. Participants were assigned to receive full ATT (248 participants) or limited control (247 participants). After adjusting for baseline imbalance of activities of daily living score, HR for median pre-institutionalisation survival was 0.84; 95% CI, 0.63 to 1.12; P = 0.20. There were no signiﬁcant diﬀerences between arms in health and social care, Group members had reduced participant-rated quality-adjusted life years (QALYs) at 104 weeks but did not diﬀer in QALYs derived from proxy-reported EQ-5D. |
| Wesselman et al., 2020 | To evaluate the effectiveness of an online lifestyle program for individuals with subjective cognitive decline and to improve the program and optimize the study procedures. | User Satisfaction and Ease of Use and the System Usability Scale. | Hello Brain is a European Project (FP7 grant no 304867) led by Trinity College Dublin. Hello, Brain comprises a website and app which are available in English French, and German. The website www.hellobrain.eu shares information and videos about the brain, brain health, and brain research. The App aims to support users to live a brain-healthy life by giving daily suggestions called 'brain buffs. | Quantitative data showed that daily advice was rated moderately useful (3.5 ±1.5, range 1-5 points). Participants (n=101, 78%) gave moderate ratings on the programs' usability (3.7±1.3, max 7), ease of learning (3.6±1.9), and satisfaction (4.0±1.5), and marginal ratings on the overall usability (63.7±19.0, max 100). CONCLUSIONS: Overall user experience of the online lifestyle program was moderate to positive. Qualitative data showed that content was appreciated and that flawless, easy access technique is essential. |
| Peterson et al., 2023 | To evaluate the feasibility, acceptability, and utility of the CarFreeMe™-Dementia intervention. | Activities of daily living/Instrumental activities of daily living, Assessment for readiness of mobility transition, Caregiver driving safety questionnaire | CarFreeMe is a seven-module psychoeducational, group-based program developed for older adults without cognitive impairment. CarFreeMe consisted of group meetings for 3–4 hr a week for 6 weeks to have discussions, speakers, and practical exercises on topics such as alternative transportation, lifestyle planning, and adjustment to loss and changes. Enrollment in CarFreeMe™ was associated with an increase in the use of alternative transportation, excursions from home, and satisfaction with transportation. | This study established initial support for CarFreeMe™-Dementia in the United States. Participants indicated the program facilitated dialogue around driving retirement and provided guidance on community engagement without driving. Respondents appreciated the program's emphasis on overall well-being, promoted through lifestyle planning and stress management. They also reported the program offered practical preparation for transitioning to driving retirement. |
| Iyer et al., 2023 | To elicit informal caregiver perceptions of tele-dementia care provided. | The interview guide informed by Fortney's Access to Care model Table: Characteristics of Study Participants Figure: The top three words to describe tele-dementia experience Five primary themes related to the caregiver's experiences | All inconsistencies between the analysts were resolved over discussion with the co-investigators in the research meetings. ATLAS.ti version 9 software was used.18 | Caregivers found tele-dementia care convenient, comfortable, stress-reducing, timesaving, and highly satisfactory. Caregivers would prefer a combination of in-person and telemedicine visits, with an opportunity to communicate with providers privately. This intervention prioritizes care for older Veterans with dementia who have high care needs and are at higher risk for hospitalization than their same-age counterparts without dementia. |
| Lindauer et al., 2017 | To evaluate the feasibility and reliability of commonly used clinical dementia assessments when administered via direct-to-home Telemedicine videoconferencing. | Clinical Dementia Rating Scale, 15-item Geriatric Depression Scale | The mode of connectivity between the study participants and the clinicians was via Cisco's Jabber TelePresence platform (Cisco, 2014). | Reliability was found to be good to excellent in all measures when used with direct-to-home telemedicine. For the most part, participants and clinicians found telemedicine to be a feasible option for assessing cognitive function and caregiver coping. These measures can be used to assess persons with AD, as well as their caregivers, across the telemedicine platform, directly to their homes. |
| Mahoney et al., 2003 | To determine the main outcome effects of a 12-month computer-mediated automated interactive voice response intervention designed to assist family caregivers managing persons with disruptive behaviors related to Alzheimer's disease. | Activities of Daily Living scale, the Instrumental Activities of Daily Living scale, the Caregiver Mastery scale | The technology consisted of an integrated telephone network system and an IVR computer network system. Caregivers used a confidential password to access the intervention system and to protect their anonymity. Caregivers dialed in from any standard touch-tone telephone and heard the narrator greet them by name, review the menu of four module options, and provide the service they requested. The telephone network operated the Personal Mailbox and Bulletin Board modules, similar to standard voice messaging systems | There was a significant intervention effect as hypothesized for participants with lower mastery at baseline on all three outcomes: bother, anxiety, and depression. Additionally, wives exhibited a significant intervention effect in the reduction of the bothersome nature of caregiving Wives who exhibited low mastery and high anxiety benefited the most from the automated telecare intervention. To optimize outcome effects, similar interventions should be tailored to match the users' characteristics and preferences. |
| Smith et al., 2023 | To examine the reliability of remote cognitive assessments compared to in-person assessments. | Montreal Cognitive Assessment | Participation in remote assessment through telephone, video conferencing, or a combination of telephone and videoconferencing | The National Alzheimer's Coordinating Center (NACC) Uniform Data Set (UDS) neuropsychological battery is being used to track cognition in par- participants across the country, but it is unknown if scores obtained through remote administration can be combined with data obtained in-person |
| Emedoli et al., 2023 | To determine if Telemedicine could ensure continuity of care. | Monthly percentage change | Telemedicine appointments are conducted remotely using video-conferencing software provided by the Institute, ensuring utmost confidentiality and privacy in a designated private room. | Resulting in an increase in the rate of telemedicine activity from 16.81% in January 2020 to 23.21% in May 2022. Peaks in telemedicine activity reached 85.64% in May 2020 and 83.65% in February 2021, there Is a notable positive correlation between telemedicine activity and the worsening of the Italian pandemic (r = 0.433, p = 0.027). Telemedicine might constitute an effective tool to promote continuity of care for patients with dementia during the pandemic. |
| Carotenuto et al., 2018 | To evaluate the reliability of the Mini-Mental State Examination and the Alzheimer's Disease Assessment Scale cognitive subscale (ADAS-cog) tests administered in hospitals by videoconference to patients with mild to moderate Alzheimer's disease. | MMSE, Activities of Daily Living, the Instrumental Activities of Daily Living, the Clinical Dementia Rating. | Administration of the MMSE and ADAS-cog tests took place through real-time videoconferencing, on both terminals, with Microsoft Skype. | This study showed no differences in the MMSE and ADAS-cog scores when the tests were administered face-to-face or by videoconference, except in patients with more pronounced cognitive deficits (MMSE<17), in which the assessment via videoconference overestimated the cognitive impairment (face-to-face, MMSE mean 13.9, SD 4.9 and ADAS-cog mean 9.0, SD 3.8; videoconference, MMSE mean 42.8, SD 12.5 and ADAS-cog mean 56.9, SD 5.5). Videoconferencing is a reliable approach to documenting cognitive stability or decline and to measure treatment effects in patients with mild to moderate dementia. |
| O'Connor et al., 2014 | To explore participant engagement and evaluations of three different targeted smartphone and Web-based dementia risk reduction tools following a four-week intervention. | Using a five-point scale, IBM SPSS Statistics Version 21 was used to conduct the analyses. | BrainyApp is a mobile device application for iPhone, iPad, and iPod Touch. This tool allows users to complete a brain health survey, which asks questions about current physical, social, and mental activity, cardiovascular health, diet, smoking, and drinking habits. The brain-heart health score achieved indicates how brain-healthy the users' current lifestyle is and particular areas for improvement are highlighted. Users can then engage in activities to improve in areas that may be increasing their dementia risk. | Results indicated that while participants across all three intervention groups reported a generally positive experience with the targeted dementia risk reduction tools, participants using the information-based website provided a more favorable evaluation across a range of areas than participants using the mobile phone app. Further research is required to investigate whether targeted dementia risk reduction tools, in the form of interactive websites and mobile apps, can be improved to provide benefits above those gained by providing static information alone. |
| Stara et al., 2021 | To evaluate the usability and gent Anne by people living with dementia. And to assess the ability of target users to use the system independently and receive valuable information from it. | Almere model, Closeness scale, System Usability acceptability of the virtual aScale | The virtual character Anne | The involved participants shared good engagement with the system, approaching the virtual agents as a companions able to support memory and enjoyment needs. Therefore, this research provides data that sustain the use of ECAs as future eHealth systems that can address the basic and higher-level needs of people living with dementia. |
| Mendez et al., 2021 | To explore factors associated with dementia caregivers' intention to adopt mHealth apps for chronic disease self-management. | Technology Acceptance Model, Zarit Burden Interview | The team recruited on the web by posting advertisements on a Johns Hopkins University online news center and social media (Google, Facebook, and YouTube) and by sending recruitment emails through a web-based research registry (ResearchMatch). | When designing mHealth app interventions for dementia caregivers with a chronic condition, it is important to consider caregivers' perceptions about how well mHealth apps can help their self-management and which app features would be most useful for self-management. Caregiving factors may not be relevant to caregivers' intention to adopt mHealth apps.MHealth strategies may overcome barriers to caregivers' self-management. |
| Lancaster et al., 2020 | To explore the little but often approach to assessment employed by the Mezurio app when prompting participants to interact every day for over a month. | Proportion compliance for Gallery Game tasks (learning, recognition, and recall) across the baseline month of assessments. The proportion of participants (n=35) completing each of the 22 Gallery Game learning tasks distributed across the baseline Mezurio assessment period. | Participants were invited to join PREVENT dementia via several routes, including the ConCERT-D and Join Dementia Research databases, as well as via the study website and social media. | Participants complied with 80% of the daily learning tasks scheduled for subsequent tests of episodic memory, with 88% of participants still actively engaged by the final task. Schedule flexibility, a clear user interface, and performance feedback are important considerations for engagement with remote digital assessment. Participants demonstrated high compliance with the schedule of daily learning tasks and were extremely positive about their experiences. Long durations of remote digital interaction are therefore definitely feasible but only when careful attention is paid to the design of the users' experience. |
| Potts et al., 2020 | To evaluate user engagement and responses to questions using InspireD, an app used for reminiscence by persons with dementia and their caregivers. Research findings can be used to use within digital health interventions. | the Mutuality Scale | The app recorded user event logs locally on each iPad using an SQLite database (a public domain structured query language database). User event log data were later collected in person from the iPads using a portable storage device. Specific activities and activity types were logged: entry (logging in), administrative activities (adding or deleting photos, videos, or music), reminiscing (viewing photos, videos, or listening to music), queries at a given instant (EMA questions), and exiting (logging out). | The main limitation of our study was the lack of generalizability of results to a larger population given the quasi-experimental design and older demographic where half of the participants were persons with dementia; however, this study shows that older people are willing to participate and engage in EMA. Based on this study, we propose a series of recommendations for app design to increase user engagement with EMA. These include presenting questions no more than once per day, after 8 PM, and only if the user is not trying to complete a task within the app. |
| Banbury et al., 2019 | Providing peer-support groups using telehealth may have the potential to develop self-sustaining peer networks for isolated caregivers of people with dementia. | the UCLA Loneliness Scale (ULS-6) selected scales from the e-Health Literacy Questionnaire | Qualitative diary logs were completed by two group facilitators, an administrator, and IT support detailing administration and IT recruitment processes. Post programme, 28 participants undertook semi-structured interviews which were thematically analysed until data saturation.11 This paper describes the digital technology implementation procedures and reports participants' and implementation team members' perceptions of its use. | Providing peer support groups using telehealth may have the potential to develop self-sustaining peer networks for isolated caregivers of PWD. |
| Williams et al., 2021 | To evaluate if Telehealth reduced caregiver depression and improved caregiver competence. | the Functional Assessment Scale (FAST) A 5-point Likert scale | Recruitment strategies were community-based and included advertisements in local magazines and newspapers, presentations at local caregiving meetings, and mass email notifications through the university community. Study Site 2 was based in an NIA-designated Alzheimer's Disease Center, where recruitment was primarily completed through electronic medical record screening using a partial HIPPA waiver. Some community recruitment was also utilized. See the published FamTechCare protocol for specific details on participant recruitment and eligibility, the protection of human subjects, intervention development, study procedures, and fidelity | Regardless of age, gender, and relationship, caregivers found the intervention acceptable and easy to use and rated the expert feedback as effective in addressing care challenges. Further adaptation may be needed for FamTechCare to be readily implemented. |
| Pot et al., 2015 | Internet interventions may offer opportunities to improve the availability and accessibility of effective interventions to reduce family caregivers' psychological distress. | Epidemiological Studies – Depression scale | All correspondence between caregiver and coach takes place within a secure, closed environment. There is no email exchange or telephone contact between them during the intervention. After each lesson, the caregiver is asked to send the ﬁnished homework exercises to the coach. If required, there is room to send a personal note as well. The coach provides feedback within three working days. In MoD, the coach is a psychologist with training in CBT and experience with family caregivers of people with dementia. | MoD reaches a wide variety of caregivers, also those aged 75+, having a relative with a recent diagnosis of dementia, or living in a care home. However, the percentage of caregivers who did not complete all eight lessons was rather high(55.7%). Among the completers (N=66;44.3%)were significantly more spouses, caregivers living in the same household, older caregivers, and those caring for somebody with another formal diagnosis than Alzheimer's disease. The evaluation showed that females rated higher on the comprehensibility of the lessons and feedback and spent less time on the lessons. |
| Pagan-Ortiz et al., 2014 | Provides bilingual information on dementia and caregiver issues. | Personal Mastery Scale (PMS) Lubben Social Network Scale, Epidemiological Studies Depression Scale. | Participants spent approximately one hour navigating the website, and were then asked questions regarding the website's appeal, usability, and communication features These questions were asked using a focus group format, and the session was conducted in Spanish. Questions regarding appeal focused on the participant's initial reactions to the site and their expectations about what it was without having a detailed explanation beforehand. Questions on usability asked participants where they would click to read about a specific question or how they could find a specific feature. | Results demonstrate the promise of this approach for enhancing the skills of caregivers of dementia patients and reducing caregivers' depressive symptomatology. Although the use of technology with Hispanic caregivers has already proven to be promising (Czaja & Rubert, 2002; Eisdorfer et al., 2003), the findings specific to the use of social media platforms with this population are a novel contribution to the caregiving literature. |
| Levinson et al., 2020 | To obtain feedback and opinions from experts and clinicians involved in dementia care and caregiver education about 1 iGeriCare and facilitators to implementing a web-based caregiver program. | Consolidated Framework for Implementation Research (CFIR) | Participants were asked to review the iGeriCare website before their interview. If they were unable to review the website before their interview, they were allowed to review it before beginning the interview. The interviewers used semistructured interview questions and asked clarifying questions as needed (Multimedia Appendix 1). A practice interview was conducted during the design of the interview guide. Participants' perceptions of iGeriCare and implementation tools were explored in particular and web-based dementia caregiver interventions and approaches to caregiver education in general. The interviewers debriefed with the broader research team after each interview to identify the emerging themes and potential areas of exploration and focus for subsequent interviews. | Study findings indicate a generally positive response to the use of internet-based interventions for dementia caregiver education. Results suggest that iGeriCare may be a useful clinical resource to complement traditional face-to-face and print material–based caregiver education. More comprehensive studies are required to identify the effectiveness and longevity of web-based caregiver education interventions and to better understand barriers and facilitators about the implementation of technology-enhanced caregiver educational interventions in various healthcare settings. |
| Dam et al., 2019 | To evaluate the internal and external validity of the Inlife intervention. | 5-point Likert scale ranging | On the Inlife platform, the primary caregiver can invite friends, family, and signiﬁcant others into three personal support circles (i.e., inner, middle, and outer circles depending on the closeness of the relationship), with diﬀerent privileges. The platform consists of the following functionalities: Proﬁle, Circles, Timeline, Calendar, Helping, Personal Messages, Care book, and Compass. These functionalities provide opportunities to share care information, messages, pictures, and requests for support. | The overall participation rate in the study was 27%. The Inlife intervention was generally well-received by the primary caregivers. Life facilitated empowerment, openness, involvement, and efficient care organization. Still, adherence was not optimal for all Inlife users. Determinants for Inlife use were identified on the level of the Inlife innovation, the users, and the socio-political context. |
| Boots et al., 2017 | To describe the process evaluation from the perspective of both family caregivers (participants) and professionals delivering the intervention (coaches) to determine internal and external validity before the effect analysis and aid future implementation. | Goal attainment scaling (GAS) | The participants are free to set their own goals. The module themes are acceptance, balance in activities, communication with family members and environment, coping with stress, focusing on the positive, insecurities and rumination, self-understanding, changing family members, and social relations and support. The participants chose 4 modules; 2 weeks were allocated for each module. However, the participants were allowed to complete the modules at their own pace by the self-management approach. | Participants and coaches were satisfied with the intervention, but adapting the content to specific subgroups, for example, younger caregivers was recommended. Implementation of the program requires more awareness of the benefits of blended care self-management programs and training in tailored self-management skills. |
| Baruah et al., 2020 | To identify the acceptability of an online training and support program for dementia caregivers in India. | Qualitative data analysis software Atlas. | The FGDs were conducted in English and each lasted 90 to 120 min. Participants were informed that FGDs would be audio-taped, prior consent was obtained, and socio-demographic details were collected. An observer also took written notes of the discussions. The moderator began with a briefing about the aim of the FGD, followed by the introduction of participants and an icebreaker session. The stem questions were open-ended and further probes were used to elicit additional information. | The study highlights the requisite components of a first-of-its-kind online training and support program in India by integrating the experiences, motivations, challenges, and expectations of caregivers and professionals involved in dementia care. |
| Mitchell et al., 2020 | To help older persons with Alzheimer's disease and related dementias remain at home while also supporting their caregiving family members. | Qualitative and quantitative methods | The remote activity monitoring (RAM) system examined in this study includes six unobtrusive motion sensors (door sensors, motion sensors, a toilet flush sensor, and a bed mattress sensor) placed in the home to detect daily activity, as well as an emergency call pendant. The sensors operate jointly, exchange information on movement or function (e.g., getting in and out of bed, opening residence doors, using the toilet), and can detect unusual activity patterns. | The integrated qualitative and quantitative data suggested that RAM technology offered ongoing monitoring and provided caregivers with a sense of security. Considerable customization was needed so that RAM was most appropriate for persons with ADRD. |
| Nunez-Naveira et al., 2016 | To support ambient assisted living-based interventions to provide support to informal caregivers of people with dementia, especially when they need to cope with their feelings of overburden or isolation. | Global Deterioration Scale (GDS) | Participants in the experimental group were provided with a link to download the Understand application on their mobile devices (Smartphone or Tablet) and also a link to use the application through the browser on their PCs. They were instructed to use the application, browse the different topics, visit the different sections, and watch the diverse media content. Participants in the control group did not use the application and maintained their usual lifestyle. | 33.3% of the caregivers were satisfied with the application and around 50% of the participants assessed it as technically and pedagogically acceptable. After using understAID the caregivers in the experimental group significantly decreased their depressive symptomatology according to the Center for Epidemiologic Studies Depression scale, but a possible benefit in their feelings of competence and satisfaction with the caring experience was also observed. |
| Boessen et al., 2017 | To test the usability and perceived value of an online platform that aims to support the communication and collaboration between family and professional caregivers of dementia. | Post-study System Usability Questionnaire | This product is an easy-to-use platform with individually tailored Cubes (i.e., applications) that access a range of services. Services of the other companies were a video conferencing tool and a tool to share care activities among (family) caregivers | The results indicate that the platform is easy to use and valuable for both family and professional caregivers. They felt better informed and prepared regarding the situation of dementia and felt supported by the more direct lines of communication within the network. Also, a broadening and deepening of the relationship between family and professional caregivers was experienced. |
| Park et al., 2020 | To evaluate the effect of a comprehensive mobile application program in managing behavior and psychological symptoms of home-dwelling patients with dementia in South Korea | the Korean Neuropsychiatric Inventory (K-NPI) Piper Fatigue Scale | The study was established to include information about the purpose of this research and about patients with dementia to improve patient factors; about methods of communication with patients with dementia, about drug and non-drug treatments, and about response methods to improve caregiver factors; and finally about environmental management methods to improve environmental factors. | The application program improved family caregivers' fatigue and burden. It also suggested there is a need to develop a wandering persons location program to improve family caregivers' stress and patients' behavioral problems in future studies. |
